# High rates of medication adherence in patients with pulmonary arterial hypertension: An integrated specialty pharmacy approach

**DOI:** 10.1371/journal.pone.0217798

**Published:** 2019-06-06

**Authors:** Nisha B. Shah, Rhonita E. Mitchell, Stephanie Terry Proctor, Leena Choi, Joshua DeClercq, Jacob A. Jolly, Anna R. Hemnes, Autumn D. Zuckerman

**Affiliations:** 1 Vanderbilt Specialty Pharmacy Services, Vanderbilt University Medical Center, Nashville, Tennessee, United States of America; 2 Huntsville Hospital, Department of Pharmacy, Huntsville, Alabama, United States of America; 3 Department of Biostatistics, Vanderbilt University Medical Center, Nashville, Tennessee, United States of America; 4 Division of Allergy, Pulmonary and Critical Care Medicine, Vanderbilt University Medical Center, Nashville, Tennessee, United States of America; Kurume University School of Medicine, JAPAN

## Abstract

Phosphodiesterase-5 inhibitors (PDE-5I) have demonstrated improvement in disease symptoms and quality of life for patients with pulmonary arterial hypertension (PAH). Despite these benefits, reported adherence to PDE-5I therapy is sub-optimal. Clinical pharmacists at an integrated practice site are in a unique position to mitigate barriers related to PAH therapy including medication adherence and costs. The primary objective of this study was to assess medication adherence to PDE-5I therapy within an integrated care model at an academic institution. The secondary objective was to assess the impact of out-of-pocket (OOP) cost, frequency of dosing, adverse events (AE) and PAH-related hospitalizations on medication adherence. We performed a retrospective cohort analysis of adult patients with PAH who were prescribed PDE-5I therapy by the center’s outpatient pulmonary clinic and who received medication management through the center’s specialty pharmacy. We defined optimal medication adherence as proportion of days covered (PDC) ≥ 80%. Clinical data including AEs and PAH-related hospitalizations were extracted from the electronic medical record, and financial data from pharmacy claims. Of the 131 patients meeting inclusion criteria, 94% achieved optimal adherence of ≥ 80% PDC. In this study population, 47% of patients experienced an AE and 27% had at least one hospitalization. The median monthly OOP cost was $0.62. Patients with PDC<80% were more likely to report an AE compared to patients with PDC≥ 80% (*p* = 0.002). Hospitalization, OOP cost, and frequency of dosing were not associated with adherence in this cohort. Patients receiving PDE-5I therapy through an integrated model achieved high adherence rates and low OOP costs.

## Introduction

Pulmonary hypertension (PH) is a chronic, progressive disease characterized by elevated pulmonary vascular pressure.[1] Pulmonary arterial hypertension (PAH) is a subgroup of PH characterized by pre-capillary PH. [2] Symptoms of PH are typically non-specific and may include shortness of breath, fatigue, angina and weakness. [3]

Phosphodiesterase-5 inhibitors (PDE-5I) are a class of medications approved for the treatment of PAH.[4] The goals of pharmacotherapy include improvement in disease symptoms and quality of life as well as prevention of disease progression.[1] Two commonly prescribed PDE-5Is are sildenafil (Revatio, Pfizer Inc., New York City, New York, USA) and tadalafil (Adcirca, Eli Lilly and Company, Indianapolis, Indiana, USA). Sildenafil demonstrated improvement in 6-minute walk distance (6MWD) and functional class as well as cardiopulmonary hemodynamics.[5] Tadalafil demonstrated improvement in 6MWD, exercise capacity and quality of life as well as a reduction in clinical worsening.[6] However, clinical effects of treatment are dependent on medication adherence. Despite proven benefit, Waxman et. al. found that less than half of patients prescribed a PDE-5I were adherent after six months, with adherence among specialty pharmacy users being significantly higher.[7] PDE-5Is and endothelin receptor antagonists, generally in combination, are used commonly for low-risk patients with PAH.[8]

To improve patient outcomes such as medication adherence, a growing number of institutions have developed integrated pharmacy practice models that implement interdisciplinary team-based care. Embedded in the clinic, pharmacists are in a unique position to mitigate barriers related to PAH therapy.[9] In this setting, pharmacists are available to assist with insurance approval, patient counseling, and management of adverse effects (AE) as well as improve coordination of care. In 2014, this model was adopted by the Vanderbilt University Medical Center (VUMC) outpatient pulmonary clinic. The pulmonary clinic collaborates with Vanderbilt Specialty Pharmacy (VSP), incorporating a clinical pharmacist and pharmacy technician as part of the healthcare team. In his or her role, the clinical pharmacist provides comprehensive medication management, patient education, and ongoing treatment monitoring as well as assistance with transitions of care. While evidence supports the use of integrated pharmacy services for the management of specialty diseases, few studies have assessed medication adherence rates or factors related to low medication adherence among patients with PAH within this model.[10, 11]

The primary objective of this study was to evaluate adherence to PDE-5I therapy for the management of World Health Organization (WHO) Group 1 PAH in patients within an integrated, multidisciplinary care model.[12] As the evaluation of factors related to low medication adherence may help guide targeted interventions in this population, our secondary objective was to assess the impact of patient out-of-pocket (OOP) cost, frequency of dosing, AE and PAH-related hospitalization on adherence rates in our study cohort.[13]

## Methods

We conducted a retrospective cohort analysis of patients with WHO Group 1 PAH prescribed a PDE-5I (sildenafil or tadalafil) by the center’s outpatient pulmonary clinic who received medication management through the center’s specialty pharmacy. Patients with less than three prescription claims during the study period were excluded given that measurement of adherence over a short period of time may lead to inaccuracy and at least three dispenses are recommended for a meaningful adherence calculation.[14] The study period was from January 1, 2014 through December 31, 2016. This time period was selected as specialty pharmacist integration occurred in 2014 and significantly impacted the clinic care model. We extracted data from specialty pharmacy claims and electronic medical records (EMR) of patients who met inclusion criteria. This study was reviewed and approved by the Vanderbilt Institutional Review Board (IRB# 170513) as an exempt study under 45 CFR 46.101 (b)(4). Data was managed using Research Electronic Data Capture (REDCap) hosted at Vanderbilt University.[15] Please refer to the S1 Table to view the specific data variables collected and analyzed.

### Participants and procedures

Patients included in the study were adults aged 18 years or older, with an International Classification of Diseases, Tenth Revision Clinical Modification (ICD-10-CM) diagnosis codes for pulmonary hypertension (ICD10 I27.0 to I27.89). The EMR was reviewed to ensure only patients with a PAH diagnosis were included.

### Outcome measures

Medication adherence was measured using proportion of days covered (PDC), defined as the total days covered by medication fills divided by the total number of days the patient was prescribed the medication during the observation period.[16] The denominator was defined by the number of days between the start and end date of the observation period. We defined the date of the first time medication was dispensed during the study period as the start of the observation period. The end date of the observation period was the last day in the study period (December 31, 2016) for those who remained on therapy for the entire duration of the study, or the date the last fill was exhausted for those who discontinued treatment before the study end date. We established the numerator by generating a supply diary of every day within the study period. At each date within the observation period (denominator), the patient was marked as possessing or not possessing the medication. If overlap occurred, we shifted the oversupply to the following days. The numerator was then the sum of the number of days covered within the supply diary. We excluded any oversupply at the end date from the numerator to ensure that the supply days did not exceed the observation window. We utilized PDC values to model factors associated with adherence as well as classify patients as adherent based on a threshold of 80% PDC. This threshold was selected given the lack of disease-state specific data correlating adherence with clinical outcomes, and its use as a benchmark for adherence in previous studies.[7] PDC was selected rather than medication possession ratio (MPR) as PDC utilizes more granular data and therefore is recommended as the preferred measure.[16]

In addition, we captured financial and insurance information from VSP claims data. We assessed patients’ insurance type (Medicare, Medicaid or commercial), use of financial assistance programs (including co-pay cards, foundation assistance or Vanderbilt-specific medication assistance), as well as monthly OOP costs incurred during the study period.

We reviewed the EMR to collect patient demographics, patient-reported AEs, and PAH-related hospitalizations. Demographic data included gender, race, age, and smoking status. We defined a hospitalization as an in-patient stay greater than 24-hours secondary to a PAH-related cause. We determined a PAH-related cause to be a chief complaint or primary diagnosis of shortness of breath, hypervolemia, hypovolemia, syncope, medication AE or heart failure that resulted in hospital admission.

### Statistical analysis

We described the study population using the number and percent for categorical variables, and means, standard deviations (SDs) and interquartile ranges (IQRs) for quantitative variables. Chi-squared tests and logistic regression were performed to test whether gender, race, smoking status, and insurance type would be associated with medication type (sildenafil vs. tadalafil) and dichotomized medication adherence with a cutoff of 80% (i.e., PDC<80% vs. PDC ≥ 80%).

Since the distribution of PDC values was highly skewed, commonly used statistical methods would not be appropriate to fit this data. Thus, we reversed the PDC values by taking 100 minus the PDC percent value as a measure of non-adherence (e.g., a PDC value of 95% corresponds with a 5% modified measure). We call it “modified non-adherence measure”, modified non-adherence measure (%) = 100 –PDC (%). The modified non-adherence measure allowed us to use a negative binomial (NB) regression model that would be useful to fit over-dispersed data with high proportion of zeros of the modified non-adherence measure (i.e., 100% PDC value). We selected candidate covariates from univariate analyses and the most important covariates were included in the final models considering the small sample size. We performed all statistical analyses using the programming language R version 3.4.3.

## Results

**Fig 1:**
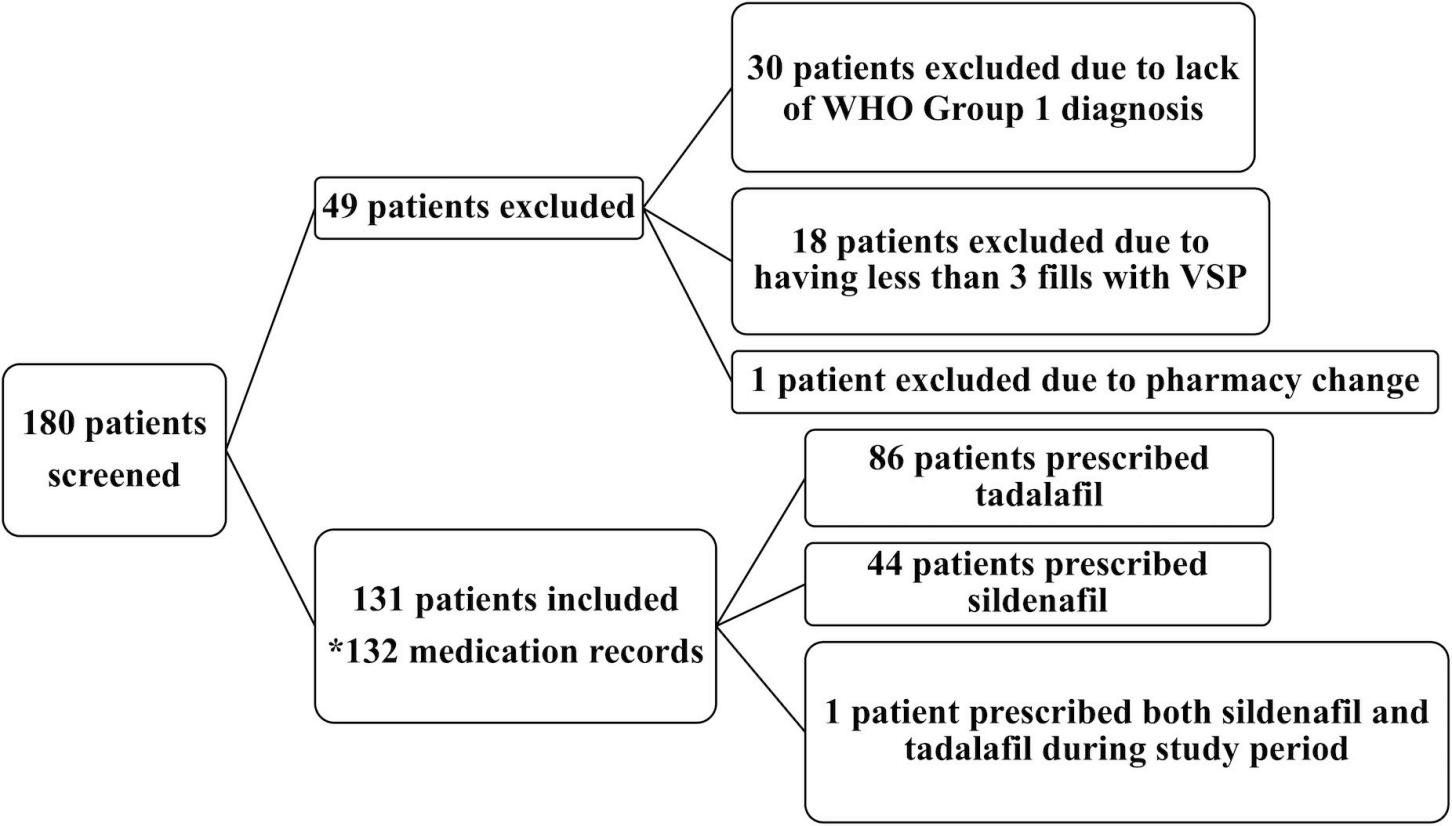
Study population. WHO = World Health Organization; VSP = Vanderbilt Specialty Pharmacy; *One patient received both sildenafil and tadalafil during study period. We utilized pharmacy claims data for both medications and created a record for both sildenafil and tadalafil for this patient.

Of the 180 patients screened, we excluded 18 patients due to having less than three prescription claims through the specialty pharmacy. We excluded an additional 30 patients due to lack of a WHO Group 1 PAH diagnosis. We excluded one patient post-evaluation due to multiple pharmacy changes during study period, which made an accurate medication adherence calculation not feasible (see Fig 1). Therefore, 131 patients were included for analysis. Of the total study cohort, 70% were female and 78% were Caucasian. Median age was 55 years (IQR: 45–62 years). More than half (62%) of the population were non-smokers and 71% had government-funded insurance. Of the 131 patients, the majority were in functional class II or III (89%; see Table 1). Half of the patients were receiving one concomitant therapy and 19% of patients were receiving two concomitant therapies.

**Table 1:** Baseline demographics for study population.

	N (%)
**Age**	
Median [IQR^a^]	55 [45–62]
**Gender**	
Female	92 (70.2)
Male	39 (29.8)
**Race**	
Caucasian	102 (77.9)
African American	28 (21.4)
American Indian/Alaska Native	1 (0.8)
**Insurance Provider**	
Commercial	38 (29.0)
Government Plan	93 (70.9)
**Smoking status**	
Never smoked	81 (61.8)
Previous smoker	38 (29.0)
Current smoker	12 (9.2)
**Phosphodiesterase-5 inhibitor**	
Sildenafil	44 (33.6)
Tadalafil	86 (65.6)
Sildenafil and tadalafil	1 (0.8)
**WHO**^**b**^ **Functional Class**	
Functional Class I	10 (7.6)
Functional Class II	51 (38.9)
Functional Class III	66 (50.4)
Functional Class IV	4 (3.0)

^a^ Interquartile range

^b^ World Health Organization

### Medication type

More than half (66%) of the study cohort received tadalafil. There was no significant difference in gender, race, smoking status, or insurance type between patients prescribed sildenafil compared to tadalafil. Of note, one patient switched therapy from sildenafil to tadalafil due to difficulty with three times a day administration of sildenafil. For this patient, we used an average PDC weighted by the number of days on each therapy, which resulted in a PDC value of 92%.

### Medication adherence

The overall average PDC for all patients was 96% (standard deviation = 0.092). Furthermore, 94% of patients achieved adherence of ≥ 80% PDC. Eight patients had a PDC value of less than 80%. Patients with PDC <80% were significantly more likely to report an AE compared to those with ≥ 80% PDC (χ^2^ = 9.48, *p* = 0.002). There was no significant difference in medication type, gender, race, smoking status, number of hospitalizations, or overall OOP cost between patients who achieved a PDC ≥ 80% compared to those who did not (Table 2).

**Table 2:** Baseline demographics by proportion of days covered (PDC).

	PDC< 80% n (% of sample)	PDC≥ 80% n (% of sample)	*P*-value^a^
**Race**			0.786
Caucasian	7 (5)	95 (73)	
African American	1 (1)	27 (21)	
Alaska Native/American Indian	0	1 (<1)	
**Smoking Status**			0.072
Never smoker	8 (6)	73 (56)	
Previous smoker	0	38 (29)	
Current smoker	0	12 (9)	
**Phosphodiesterase-5 inhibitor**^b^			0.576
Sildenafil	2 (2)	43 (33)	
Tadalafil	6 (5)	81 (61)	
**Concomitant therapy**			
Endothelin receptor antagonist	4 (3)	64 (49)	0.91
Prostanoid	4 (3)	36 (27)	0.22
Calcium channel blocker	0	6 (5)	0.52
Prostacyclin receptor agonist	0	5 (4)	0.56
**Adverse Event**			0.002
Yes	8 (6)	54 (41)	
No	0 (0)	69 (53)	
**Hospitalization**			0.910
Yes	2 (2)	33 (25)	
No	6 (5)	90 (69)	
**Monthly out-of-pocket cost**			0.838
$0	4 (3)	45 (34)	
>$0-$10	3 (2)	55 (42)	
>$10–100	1 (1)	17 (13)	
>$100	0 (0)	6 (5)	
**Financial assistance**			0.062
Yes	7 (5)	66 (50)	
No	1 (<1)	57 (44)	

^a^ statistical significance tested by Pearson’s chi-squared test

^b^ n = 132 as one patient received both sildenafil and tadalafil.

**Fig 2:**
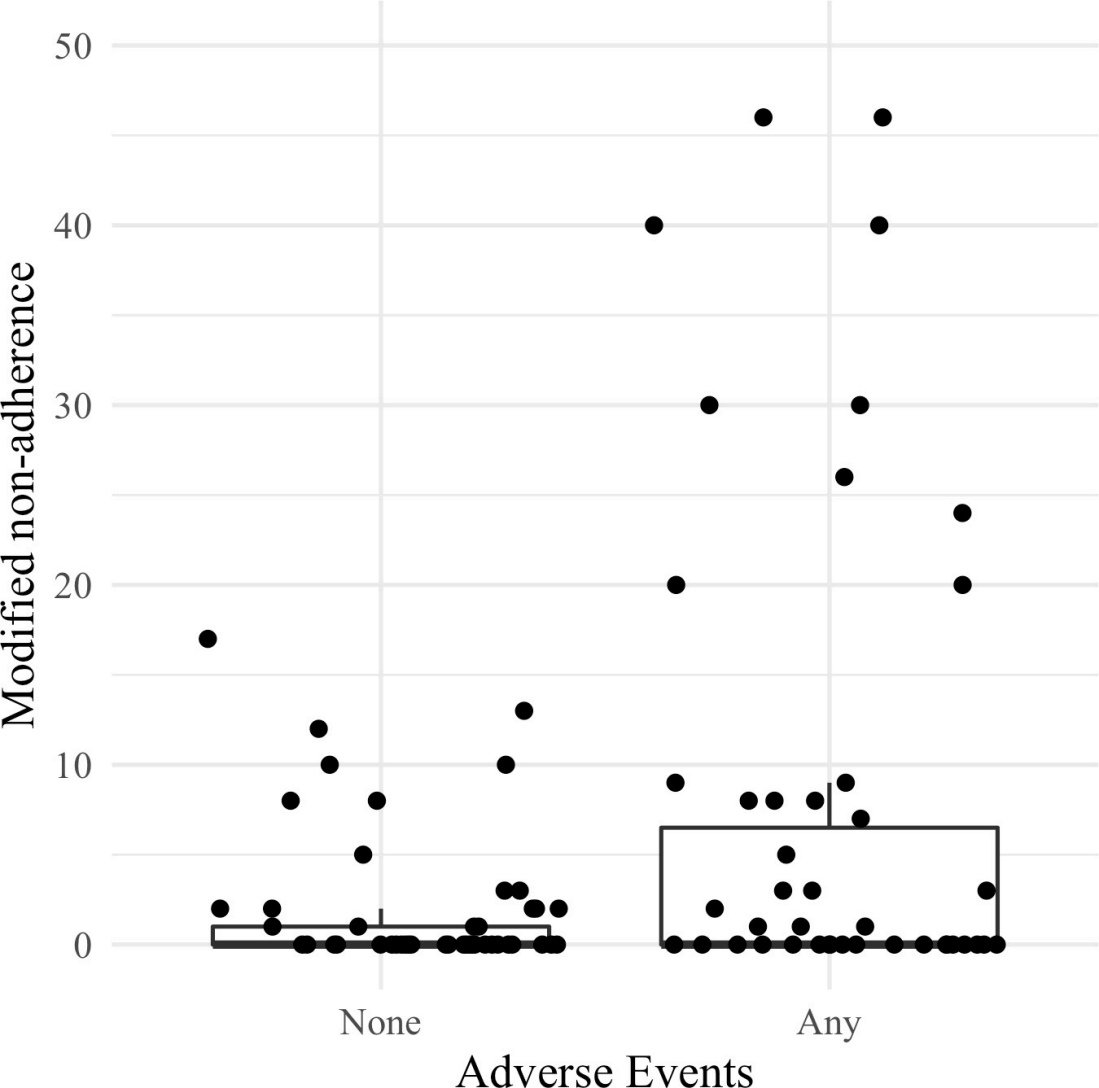
Boxplot illustrating modified non-adherence by patients reporting an adverse event. Patient data represented using jittered dots. Due to a high proportion of patients with 0% modified non-adherence (i.e., 100% raw PDC score), the 25th percentile and the median both overlap at 0%. The top of the box represents the 75th percentile of the data. The y-axis only extends up to 50% because the highest modified non-adherence value was 46%. Modified non-adherence was calculated as 100 –raw PDC value in percentage (%).

Fig 2 shows the distribution of the modified non-adherence by a group of patients who experienced an AE versus those who did not, where the distribution was shifted to higher values of the modified non-adherence for patients who experienced an AE. This apparent trend was confirmed in the NB regression analysis; patients reporting an AE were 4.2 times more likely to be non-adherent to PDE-5I therapy compared to patients who did not experience an AE (incidence rate ratio = 4.2; 95% CI:1.6 to 11.1; *p* = 0.004).

### Adverse events

In this study population, 47% of patients experienced an AE during the study period. The mean PDC of the subset of patients reporting an AE was 94% (standard deviation = 0.123). The most common AE was headache (24%) with seven of the eight patients who had <80% PDC reporting a headache while on therapy. Other AEs included reflux (11%), diarrhea (11%), leg pain (6%) and nausea/vomiting (5%). Occurrence of an AE was not associated with medication type, age, race, gender, or smoking status in this cohort. In addition, there was no significant difference in AE between patients on sildenafil compared to those on tadalafil (*p* = 0.23).

### Hospitalizations

While the majority (73%) of patients in this study did not have a PAH-related hospitalization during the two-year period, 24% of the study population had 1 to 2 hospitalizations and 3% had ≥ 3 hospitalizations. Among the total 35 hospitalizations, the majority of patients (69%) were in functional class III. The most common chief complaint upon admission was shortness of breath (54%). One patient experienced diarrhea that led to hospitalization. From unadjusted analyses, females were less likely to be hospitalized when compared to males (unadjusted OR = 0.438, 95% CI 0.194–0.988, *p* = 0.047).

### Medication cost

The median monthly OOP medication cost was $0.62 (Range: $0 to $354.71; IQR: $0.00-$5.83), with 37% of patients having no monthly OOP cost. Of those who incurred a monthly OOP cost, 44% of patients paid less than $10 per month. Additionally, 14% of patients had a monthly OOP cost greater than $10 but less than $100, and 5% of patients had an OOP of greater than $100 per month. Patients with commercial insurance had a significantly higher OOP cost than patients with non-commercial insurance (median OOP $54 compared to $4.20, *p*<0.001). However, we found that monthly OOP cost did not influence PDC within our study (*p* = 0.84).

### Financial assistance

More than half of patients (56%) used one or more financial assistance programs. Nineteen percent of patients received assistance through a manufacturer copay card, 34% of patients received assistance from a foundation, and 16% used the Vanderbilt Medication Assistance Program. More than half of Medicare beneficiaries as well as the majority of patients with commercial insurance required financial assistance (53% and 71%, respectively). Of those patients receiving assistance, 81% were on tadalafil and 19% were on sildenafil or Revatio. There was no difference in OOP cost between patients with financial assistance compared to those without it (*p* = 0.86).

## Discussion

### High adherence in integrated pharmacy practice model

Overall, patients in this study cohort achieved high medication adherence (mean PDC of 96% for both sildenafil and tadalafil) over a two-year period. This value exceeds the adherence rates observed by Waxman et al. among specialty pharmacy users over a 6-month period (63.2% for Adcirca and 66.5% for Revatio).[7] The previous study found higher medication adherence in patients prescribed PDE-5I therapy by a pulmonologist compared to a primary care physician (39.2% vs. 29.2%) among retail pharmacy users.[7] Our study supports the finding that patients receiving care in a specialty disease clinic with an integrated team are able to maintain high adherence to therapy.

Using healthcare claims data, Copher and colleagues reported similarly high medication adherence to sildenafil with an average MPR of 0.96 in 247 patients.[17] However, this study consisted of primarily commercially-insured patients (86%) as compared to a high proportion of patients with government insurance in our study. Additionally, researchers utilized MPR to measure adherence whereas our study utilized the more conservative measure of PDC.[16]

### Impact of adverse events and hospitalizations

Nearly half of the study patients experienced a treatment-related AE, with headache being the most commonly reported. The occurrence of headache in this study cohort was at a similar rate compared to previous trials.[5, 6] Due to our integrated model, we were able to link pharmacy claims data with the EMR to assess for a possible relationship between medication adherence and occurrence of AEs or hospitalizations. Although patients experiencing an AE were 4.2 times more likely to be non-adherent (*p* = 0.004), this subset still achieved high medication adherence (mean PDC of 94%). Within the VSP model, patients receive counseling on AEs prior to initiation and at regular follow-up intervals. This model of frequent patient engagement permits identification and resolution of AEs in an effort to avoid a lapse in therapy.

The impact of PAH-related hospitalization on medication adherence was a novel covariate analyzed. Data from the Registry to Evaluate Early and Long-term PAH Disease Management (REVEAL), a multicenter, observational, US-based registry designed to evaluate the longitudinal course of PAH, found that 56.8% of patients experienced a hospitalization and 52.4% of those hospitalizations were secondary to PAH.[18, 19] A retrospective administrative claims database study found that about 1 in 3 patients experienced a hospitalization in the first 6 months after treatment initiation.[20] Our study cohort also reported a high incidence of hospitalization, with 27% of patients experiencing at least one PAH- related hospitalization. Importantly, the mean PDC value remained high at 97% in this subset. Within an integrated model such as VSP, the clinical pharmacist is in a unique position to assist with transitions of care. Pharmacist involvement in transitions of care has demonstrated improvement in patient satisfaction, Hospital Consumer Assessment of Healthcare Providers and Systems scores, and prevention of post-discharge AEs.[21–23] It is possible that hospitalization did not negatively influence adherence due to the increased coordination of care made possible by our integrated model.

Previous studies have shown that greater length of time on targeted therapy and use of a compliance aid may be negatively associated with adherence in patients on PAH therapy.[13] Although our study did not assess these factors, the study duration was a 2-year period suggesting that this model is able to achieve sustained high medication adherence rates.

### Influence of out-of-pocket cost and dosing frequency

Given previous findings that frequency of dosing and medication cost may influence adherence to PDE-5I therapy, we examined each of these covariates to determine their potential effect on PDC.[7, 13] Our study found no difference in medication adherence between patients prescribed sildenafil (three times daily administration) or tadalafil (once daily administration) with both groups achieving a PDC of 96%; thus, frequency of dosing did not have an impact on medication adherence in our integrated model. One possible explanation for this is comprehensive medication management provided within a high-touch specialty pharmacy model, resulting in maintenance of adherence despite increased dosing frequency. Our results support the finding by Waxman et al. that, while adherence to Adcirca was higher than adherence to Revatio for retail pharmacy users, the rates of adherence were similar between the two groups for specialty pharmacy users (63.2% vs. 66.5%, *p* = 0.348).[7]

When comparing specialty pharmacy users paying different OOP co-payments, Waxman, et al. found a lower rate of adherence in the group paying $251+ compared to the $0-$50 group (53.9% vs. 67.4%, *p*<0.05).[7] In contrast, higher monthly OOP cost in this study cohort was not associated with decreased medication adherence, even in patients with OOP cost exceeding $100 per month. However, it is difficult to correlate high OOP with low medication adherence in this cohort because most patients in our study (80%) had either $0 or $0-$10 OOP cost per month and only 5% of patients had an OOP of greater than $100 per month. Therefore, it is possible the high copayments seen in the previous study represent a population that did not have the level of financial assistance provided to VSP patients. Ensuring affordability of treatment is an integral role of the VSP clinical pharmacist. The clinical pharmacist works closely with the patient to address financial barriers such as change of insurance, high copayment, and coverage gaps. As the majority of patients in our cohort required financial assistance, it is likely that this step was fundamental to maintaining adherence.

### Role of the specialty pharmacy

Given the high medication adherence rates seen in this integrated model, we believe it vital to describe the care model provided to the cohort studied. Prior to the integration of a clinical pharmacist, provider burden, prior authorization requirements, and high OOP costs often delayed or interrupted treatment. In 2014, VSP collaborated with the clinic to incorporate a clinical pharmacist and pharmacy technician as patient care partners. These embedded services has streamlined medication access issues and increased coordination of care. Of note, specialty medications are not auto-filled within our model. Rather, a pharmacy technician and/or pharmacist must speak to the patient monthly prior to a refill shipment. Further, the clinical pharmacist provides patient education during clinic visits and collaborates with providers to determine cost-effective, optimal treatment regimens.

### Limitations of the study

As a single-center, nonrandomized, retrospective cohort study, our findings may not be generalizable to all patients with PAH. In addition, because researchers only had access to VSP claims data, a comparator arm was not feasible. Furthermore, researchers could only capture AEs and hospitalizations if documented in the EMR. While it is an objective measure, PDC reflects prescription refills but not actual medication consumption.

Our sample size was small relative to a highly skewed distribution of adherence measures; hence, it is possible that the study was not be able to identify potentially important covariates associated with adherence due to insufficient power. Lastly, patients were required to have at least three prescription claims for inclusion to allow for an appropriate observance of patient adherence behavior.

### Future directions

Longitudinal studies conducted within an integrated pharmacy practice model would provide meaningful information about long-term relationship between medication adherence and health outcomes. Furthermore, studies comparing integrated specialty pharmacy models such as VSP with non-integrated models would be useful to assess the value-add of this care delivery model.

Although patients on combination therapy were not excluded from this study, we could not evaluate the effect of combination therapy on medication adherence as we did not have access to pharmacy claims data for non-PDE5I agents. Combination therapy is a strategy commonly used in PAH patients and has previously been noted to negatively impact adherence rates compared to patients on monotherapy.[13] Further studies assessing medication adherence in patients on combination PAH therapy would be valuable. While the integrated specialty pharmacy model resulted in high adherence to PDE-5I therapy, it would be useful to determine what effect, if any, this model has on adherence to dual and triple therapy.

## Conclusions

Patients receiving care within the high-touch, integrated VSP model maintained high rates of medication adherence over a two-year period. Furthermore, previous predictors of low medication adherence such as increased dosing frequency and medication cost did not have a negative impact on adherence in this cohort.

## Supporting information

S1 Table Data variables. (DOCX)
