# Supplementary material for: High rates of medication adherence in patients with pulmonary arterial hypertension: An integrated specialty pharmacy approach
Source: PLoS One. 2019 Jun 6;14(6):e0217798. doi: 10.1371/journal.pone.0217798 (PMC6553732; doi:10.1371/journal.pone.0217798)
Supplement: S1 Table — (DOCX) [file pone.0217798.s001.docx]

| **High Rates of Medication Adherence in Patients with Pulmonary Arterial Hypertension: An Integrated Specialty Pharmacy Approach**  **Supporting information: Study variables** |
| --- |
| Age |
| Gender (male/female) |
| Race   - Caucasian - African American - American Indian/Alaska Native - Asian - Native Hawaiian or other Pacific Islander - Unknown/not reported |
| PDE-5 Inhibitor   - Sildenafil - Tadalafil |
| Smoking status   - Never smoker - Current smoker - Previous smoker |
| Insurance type   - Commercial - Government (Medicare/Medicaid) |
| Financial assistance (yes/no) |
| Type of financial assistance   - Co-pay card/manufacturer assistance - Vanderbilt medication access program - Foundation assistance |
| Concomitant medication   - Endothelin receptor antagonist - Prostanoid - Soluble guanylate cyclase stimulator - Calcium channel blocker - Prostacyclin receptor agonist |
| Out-of-pocket cost |
| Adverse event (yes/no) |
| Adverse event, type   - Headache - Nausea and vomiting - Diarrhea - Leg pain - Fatigue - Dizziness |
| Adverse event, other (free text response) |
| Hospitalization (yes/no) |
| Number of hospitalizations |
| Chief complaint for hospitalization   - Shortness of breath - Hypervolemia - Hypovolemia - Syncope - Medication side effect - Heart failure |
| Chief complaint for hospitalization, other (free text) |
| Prescription data (used to calculate proportion of days covered)   - For *each* prescription dispensed for *each* patient:   - The number of days since the first dispense   - The amount of medication supplied |
| Duration of observation period   - Number of days between first dispense and date the last dispense is exhausted or end of study period (*earlier of the two*) |
| Status at end of study period   - Living - Deceased |
